# Dyslipidemia in Patients with Type 2 Diabetes Mellitus in a Tertiary Care Centre: A Descriptive Cross-sectional Study

**DOI:** 10.31729/jnma.6278

**Published:** 2021-04-30

**Authors:** Ram Kumar Mehta, Puru Koirala, Ram Lala Mallick, Surya Parajuli, Rajneesh Jha

**Affiliations:** 1 Department of Internal Medicine, Birat Medical College Teaching Hospital, Budhiganga, Morang, Nepal; 2 Department of Biochemistry, Birat Medical College Teaching Hospital, Budhiganga, Morang, Nepal; 3 Department of Community Medicine, Birat Medical College Teaching Hospital, Budhiganga, Morang, Nepal

**Keywords:** *dyslipidemia*, *prevalence*, *T2DM*

## Abstract

**Introduction:**

Dyslipidemia is highly prevalent among type 2 diabetic patients. It increases the risk of atherosclerosis and consequent mortality in diabetic patients. The aim of this study was to find out the prevalence of dyslipidemia among type 2 diabetic patients.

**Methods:**

This was a descriptive cross-sectional study in 355 type 2 diabetic patients at tertiary care hospital from 15th May, 2020 to 15th November, 2020 after taking ethical clearence from Institutional Review Committee (Reference no. IRC-PA-052/2077-78). Convenience sampling was done. Demographic and lipid profile variables were recorded based on the structured questionnaires. Data were analyzed by Statistical Package for the Social Sciences version 20. Point estimate at 95% Confidence Interval was calculated along with frequency and percentage for binary data.

**Results:**

Out of total 355 cases of type 2 Diabetes mellitus, prevalence of dyslipidemia was 224 (63.1%). It was more prevalent in male 145 (69.4%) than female 79 (54.1%). Increased Low density Lipoprotein (94.2%) was the most prevalent type followed by mixed dyslipidemia (91.1%).

**Conclusions:**

Dyslipidemia was common among type 2 diabetic patients and was higher in male gender, older age, obesity and longer duration of diabetes. Hence type 2 diabetic patient should undergo the routine monitoring of blood sugar and lipid profile so that any abnormalities can be identified and preventive measures along with interventions can be initiated at the earliest.

## INTRODUCTION

Dyslipidemia is one of the major modifiable risk factor for the development of type 2 diabetes mellitus (T2DM), atherosclerosis, stroke and cardiovascular diseases.^1^ The occurrence of T2DM exists as the third major non-communicable disease in Nepal.^2^

In Nepal, prevalence of dyslipidemia is 63.8% in eastern, 61.0% in central and 90.7% in mid-western region.^3^ Dyslipidemia is one of the important risk factor for vascular complications in diabetic patients. It increases free fatty acid flux secondary to insulin resistance and aggravated by increased inflammatory adipokine.^4^ T2DM increases the risk of cardiovascular disease (CVD) several fold.^5^

Early detection and proper management of dyslipidemia in diabetic patient helps in prevention of diabetes related complications.^6^ Thus, this study aimed to determine the prevalence of dyslipidemia among Nepalese type 2 diabetic population residing in this region.

## METHODS

This is a descriptive cross-sectional study conducted at Birat Medical College Teaching Hospital, Nepal from 15th May 2020 to 15th November 2020. A total of 355 patients with Type 2 Diabetes Mellitus (Age >30 years) who attended medicine Out Patient Department were enrolled based on convenience sampling technique.

Prior to the study, ethical clearance was taken from Instutional Review committee (IRC-PA-052/2077-78). Written consent was taken from all type 2 diabetic patients of age more than 30 years and willing to be enrolled in the study. Patient presenting in diabetic ketoacidosis, hyperglycemic hyperosmolar state and denied to give written consent were excluded from the study. Sample size was calculated using the formula,

$$\begin{array}{ll} \text{n} & ={\text{Z}}^{\text{2}}\times \text{p}\times \text{q}/{\text{e}}^{\text{2}}\\ & ={\left(1.96\right)}^{2}\times 0.638\times 0.362/0.05^{\text{2}}\\ & =355 \end{array}$$

where,

n = required sample size
Z = 1.96 at 95% Confidence Interval (CI)
p = prevalence of the dyslipidemia, 63.8%^3^
q = 1-p
e = margin of error, 5%

Sample size of 355 was considered in this study. All patients who met inclusion criteria were sent for lipid profile assay in department of biochemistry. Sample was collected in overnight fasting state. All participants were asked relevant questions to note demographic information, co-morbid conditions based on prestructured questionnaires.

For serum lipids. National Cholesterol Education Programme Adult Treatment Panel III (NCEP-ATPIII) guideline was referred.^7^ According to NCEP-ATPIII guideline hypercholesterolemia-serum total cholesterol level ≥200 mg/dl; hypertriglyceridemia-serum TG level ≥150 mg/dl; low HDL-C level ≤40 mg/ dl for men and ≤50mg/dl for women; high LDL-C level ≥100 mg/dl. Dyslipidemia is defined by presence of one or more than one abnormal serum lipid concentration. Mixed dyslipidemia is defined by presence two or more than two abnormal value of above mentioned lipid parameters. T2DM was designated to the patients who were already diagnosed by a physician and under treatment and/or who had plasma glucose levels above the cut off values recommended by American Diabetes Association (ADA) criteria.^8^

Data were entered and analyzed using the Statistical Package for the Social Sciences version 20.0 (SPSS 20) for Windows. Demographic variables were analyzed using descriptive statistics. Data were collected throughout the study period to meet the sample size for the study.

## RESULTS

The prevalence of dyslipidemia among type 2 diabetics is 224 (63.1%). Similarly, prevalence of dyslipidemia among male is 145 (69.4%) and female is 79 (54.1%). Out of 355 patients, 209 (58.9%) were male and 146 (41.1%) were female. Mean age of participants was 53.10±13.74 years (Table 1).

**Table 1:** Socio-demographic details of participants (n=355).

Variables	Male	Female	Total
n (%)	209 (58.9)	146(41.1)	355(100)
Mean age (years)	53.99±13.86	51,82± 13.50	53.10±13.74
Age group (years)			
30-44	61 (29.2%)	49 (33.6%)	110(31%)
45-60	74 (35.4%)	47 (32.2%)	121 (34.1%)
60-74	62 (29.7%)	44 (30.1%)	106 (29.9%)
>75	12 (5.7%)	6(4.1%)	18(5.1%)
BMI (kg/m^2^)			
<25	95 (45.5%)	75 (51.4%)	170 (47.9%)
>25	114(54.5%)	71 (48.6%)	185 (52.9%)
Mean Waist Circumference (cm)	90.74	88.34	89.75
Diet			
Vegetarian	15 (7.2%)	38 (26%)	53(14.9%)
Non vegetarian	194 (92.8%)	108 (74%)	302 (85.1%)
Smoking			
Current smoker	58 (27.8%)	30 (20.5%)	88 (24.8%)
Ex-smoker	54 (25.8%)	18(12.3%)	72 (20.3%)
Non-smoker	97 (46.4%)	98 (67.1%)	195 (54.9%)

Abbreviation: BMI; body mass index

Most common type of dyslipidemia among type 2 diabetics was high LDL was 211 (94.2%) followed by mixed dyslipidemia 204 (91.1%) (Table 2).

**Table 2:** Prevalence of abnormal lipid profile among T2DM patients (n=224).

	n (%)
Dyslipidemia	224 (63.1)
Male	145 (69.4)
Female	79 (54.1)
High total cholesterol	184 (82.1)
Low HDL	105 (46.9)
Isolated low HDL	8 (3.6)
High LDL	211 (94.2)
Isolated high LDL	7(3.1)
High triglyceride	165 (73.7)
Isolated high triglyceride	5 (2.2)
Mixed dyslipidemia	204 (91.1)

Prevalence of dyslipidemia was high in patients with high BMI and high LDL was the most prevalent one 173 (93.5%). Prevalence of single and mixed dyslipidemia was higher in patients with longer duration of diabetes (Table 3).

**Table 3:** Prevalence of single and mixed dyslipidemia based on various characteristic of diabetic patients (n=355).

		High TC	Low HDL	High LDL	High TG	Mixed
		n (%)	n (%)	n (%)	n (%)	n (%)
Gender						
Male	209	116(55.5)	50 (23.9)	136(65.1)	108(51.7)	130 (62.2)
Female	146	68 (46.6)	55 (37.7)	75(51.4)	57 (39)	74 (50.7)
Age group						
30-44	110	13 (11.8)	13 (11.8)	20 (18.2)	17 (15.5)	17 (15.5)
45-59	121	60 (49.6)	18 (14.9)	69 (57.0)	49 (40.5)	66 (54.5)
60-74	106	94 (88.7)	59 (55.7)	104 (98.1)	82 (77.4)	103 (97.2)
>75	18	17 (94.4)	15 (83.3)	18 (100)	17 (94.4)	18 (100)
BMI						
<25	170	34 (20)	20(11.8)	38 (22.4)	33 (19.4)	38 (22.4)
>25	185	150 (81.1)	85 (45.9)	173 (93.5)	132 (71.4)	166 (89.7)
Duration of Diabetes (years)						
<5	149	29(19.5)	25(16.8)	37 (24.8)	30 (20.1)	34 (22.8)
5-10	65	39 (60.0)	9(13.8)	44 (67.7)	28(43.1)	43 (66.2)
10-15	51	32 (62.7)	20 (39.2)	41 (80.4)	32 (62.7)	38 (74.5)
>15	90	84 (93.3)	51 (56.7)	89 (98.9)	75 (83.3)	89 (98.9)
Diet						
Vegetarian	53	30 (56.6)	18 (34.0)	32 (60.4)	24 (45.3)	31 (58.5)
Non vegetarian	302	154 (51.0)	87 (28.8)	179 (59.3)	141 (46.7)	173 (57.3)

## DISCUSSION

The aim of our study was to determine the prevalence of dyslipidemia among type 2 diabetic patients residing in Eastern Nepal. Majority of patients were male, male to female ratio was 1.4:1. Most of the participants were non-vegetarian (85.1%). Similarly more than half of patients were having higher BMI (52.9%) which is similar to study conducted in Thailand.^9^ Changes in lifestyle such as consumption of western style diets which include high calorie foods with increased carbohydrate, fat, and red meat consumption and low fiber diet have contributed to the increased prevalence of obesity, metabolic syndrome and T2DM.^10^

There was high prevalence of dyslipidemia in patients with diabetes (63.1%) which is similar to study done by Das et al.^11^ However this was slight lower than other study reported from other part of the country.^12^ Our study demonstrated higher prevalence of dyslipidemia among male than female i.e. 69.4% and 54.1% respectively which is comparable to similar study done at tertiary center in this region.^11^ When focusing on the subtypes of dyslipidemia, most prevalent was high LDL (94.2%) followed by mixed dyslipidemia (91.1%), high total cholesterol (82.1%), hypertriglyceridemia (73.7%) and low HDL (46.9%). High LDL was most prevalent dyslipidemia in both male and female which was 65.1% and 51.4% respectively. These findings are similar to that of reports from Nepal and elsewhere.^3,13^ Atherogenic dyslipidemia was present in most of the patients which might be due to insulin resistance and is exaggerated by the hyperglycemic state and lipotoxicity; all of these factors can lead to increased risk for atherosclerotic cardiovascular disease.^14^

Dyslipidemia was more prevalent in patients having higher BMI and high LDL was the most prevalent (93.5%) followed by mixed dyslipidemia (89.7%). This is in accordance to the report from different regions of Nepal.^3^ Higher BMI is directly associated with abdominal obesity which is one of the important risk factor for cardiovascular events. Presence of visceral adipose tissue can lead to insulin resistance as well as dyslipidemia.^15,16^ Prevalence of dyslipidemia was shown to be increasing with ageing in our study and most common abnormality was high LDL, which is similar to study done by Pokharel, et al.^3^ As age increases there will be reduced expression of hepatic LDL-C receptors leading to increased total cholesterol and LDL level due to impaired clearance from plasma.^17^ Prevalence of dyslipidemia was shown to be more in patient with longer duration of diabetes and most common lipid abnormality was high LDL. This is supported by other study as well.^18^ The exact mechanism by which altered lipid profile is more deranged with disease duration is not very well understood.

This is a small cross-sectional hospital based study representing diabetic patients from the eastern part of Nepal. It did not analyze the effect of lipid lowering treatment in dyslipidemic patients. Glycemic control has not been assessed in this study. The findings of this study, therefore, should be interpreted within context, and may not be generalized to the whole diabetic patients.

## CONCLUSIONS

Our study showed high prevalence of dyslipidemia among type 2 diabetic patients. Dyslipidemia was higher in male gender, older age, obesity and longer duration of diabetes. These findings should raise awareness regarding prevalence of dyslipidemia among type 2 diabetics to healthcare providers and will help in management of dyslipidemia. Indeed, efforts should be made to increase awareness to public about diabetic dyslipidemia, benefits of change in lifestyle and regular intake of medication and thus decreasing the prevalence of dyslipidemia among diabetic patients.
